# Promotion Effect of Steam on Hydrogen and Oxygen Separation in Electrochemical Membrane Reactor: Investigation Under the Aromatization Reaction Temperature Window

**DOI:** 10.3390/membranes16080260

**Published:** 2026-07-31

**Authors:** Lihui Wang, Shao Zhang, Mingming Wang, Zhigang Wang, Xiaoyao Tan

**Affiliations:** 1 State Key Laboratory of Advanced Separation Membrane Materials, Department of Chemical Engineering, Tiangong University, Tianjin 300387, China; 2020020045@tiangong.edu.cn (L.W.);; 2 Hebei Industrial Technology Research Institute of Membranes, Cangzhou Institute of Tiangong University, Cangzhou 061000, China

**Keywords:** perovskite membrane, water electrolysis, hydrogen permeation, oxygen permeation, external circuit, membrane reactor

## Abstract

Methane aromatization mainly proceeds at 650–750 °C. Simultaneous separation of hydrogen and oxygen can boost conversion efficiency and mitigate catalyst coking, yet most non-electrochemical membrane reactors fail to achieve synchronous hydrogen–oxygen separation within this temperature range. Accordingly, an electrochemical membrane reactor is adopted in this work, and steam is introduced to improve gas separation efficiency. BZCY hollow fiber membranes with mixed proton and oxygen ion conductivity are selected as the research material, and the influences of three distinct steam feeding modes (anode side only, cathode side only, simultaneous feeding on both sides) on H_2_ and O_2_ permeation and separation are systematically investigated. Experimental results reveal that steam humidification significantly enhances the permeation fluxes of hydrogen and oxygen. Notably, such promotional effect strongly depends on the steam feeding location. At 700 °C and 1.5 V, the hydrogen permeation flux increases to 1.469 mL⋅min^−1^⋅cm^−2^, in sharp contrast to 0.189 mL⋅min^−1^⋅cm^−2^ under dry atmosphere. Meanwhile, the oxygen permeation flux reaches 0.824 mL⋅min^−1^⋅cm^−2^ at 700 °C with steam, which is approximately four times that under dry conditions. This study verifies the intrinsic H_2_ and O_2_ permeation capability of electrochemical membrane reactors and the remarkable promotion effect originating from steam, facilitating further practical applications of such membrane reactors in methane aromatization.

## 1. Introduction

Direct methane dehydroaromatization (CH_4_ → C_6_H_6_ + 9H_2_, ΔGr = +433 kJ⋅mol^−1^, ΔHr = +531 kJ⋅mol^−1^) [1] is a promising catalytic technology for converting methane into high-value chemicals such as benzene. Nevertheless, this reaction suffers from low methane conversion and rapid catalyst deactivation caused by carbon deposition, which greatly hinders its industrial application. To effectively improve the performance of methane aromatization, researchers have integrated catalysts with gas separation membrane reactors. Such reactors can realize in situ hydrogen removal to raise methane conversion or introduce oxygen into the reaction system to alleviate catalyst deactivation [2,3]. However, the commonly used catalysts operate stably only within 650–750 °C and tend to deactivate above 800 °C [4,5]. Meanwhile, membrane reactors working under non-electrochemical modes exhibit poor separation performance in this temperature range. Accordingly, electrochemical membrane reactors, which can be coupled with catalysts, deliver excellent permeation performance and match the reaction temperature window, possess remarkable technical advantages.

High temperature proton-conducting ceramic membranes (HTPCMs) of the type based on doped SrCeO_3_, BaCeO_3_, CaZrO_3_ or SrZrO_3_ perovskite oxides have been extensively studied over the past few decades for their promising applications in fuel cells, gas sensors, hydrogen pumps/separators, and catalytic membrane reactors [6,7,8,9,10,11,12,13]. The chemical composition of these perovskites may be written as AB_1−x_M_x_O_3−δ_, where the A^−^ and B^−^ site are occupied with Ba^2+^/Sr^2+^/Ca^2+^ and Ce^4+^/Zr^4+^ respectively; M is the trivalent dopant like rare earth elements; x the dopant fraction, which is less than its upper limit to form solid solution (usually <0.2); and δ is the oxygen deficiency per perovskite oxide unit cell. As a typical example, BaCe_1−x−y_Zr_x_Y_y_O_3−δ_ composite co-doped with zirconia and yttrium oxide to achieve both high ionic conductivity and high stability has been well applied in recent years as the electrolyte to construct proton ceramic fuel cells with high power density [14,15,16,17,18] and electrochemical reactors for methane conversion into aromatics or ammonia synthesis at atmospheric pressure [19,20,21]. The oxygen vacancies generated by the doping of aliovalent cations allow the HTPC perovskite with oxygen ion conduction at elevated temperatures. Meanwhile, the perovskite oxides also may take protons from the water/hydrogen molecules in the surroundings at high temperature to form interstitially mobile protons, leading to proton conduction [22,23,24]. The individual conductivity of the perovskite, which is related to the concentration and mobility of the charged defects, is not only determined by the oxide composition but also affected by the surrounding gas atmosphere and temperature [25,26,27]. When an electrical gradient or an oxygen/hydrogen partial pressure gradient is applied to the HTPCMs at an elevated temperature, the oxygen and/or hydrogen permeation through the membrane may take place, with the flux depending on gas compositions in the permeation cell [28,29,30,31]. The water vapor often existing in both the fuel cell and the membrane reactor systems plays an important and complex role in oxygen/hydrogen permeation [32]. On the one hand, the oxygen in water molecules may be incorporated into the oxide vacancies of the perovskite, while two protons are released at the same time to the oxygen lattice [33,34]. As a result, both the oxide and the protonic conductivity of the perovskite may be affected, leading to a variation in the permeation behaviors of the HTPCMs. Furthermore, steam transport may take place in the perovskite via the ambipolar diffusion of protonic defects and oxygen ion vacancies [34,35]. On the other hand, the splitting of water vapor under the applied voltage also contributes to the overall flux of hydrogen and oxygen through the membranes [36,37].

In this study, BZCY perovskite hollow fiber membranes were prepared by phase inversion and the sintering technique. The configuration of hollow fibers facilitates high-temperature sealing connections, simplifying the operations for high-temperature testing [38]. We constructed various permeation test setups and investigated the enhancement rules of steam on hydrogen and oxygen permeation in electrochemical membrane reactors at 600–800 °C. This work provides a basis for the development of electrochemical catalytic membrane reactors applicable to methane aromatization.

## 2. Results and Discussion

### 2.1. Characterization of BZCY Hollow Fiber Membrane

**Figure 1:**
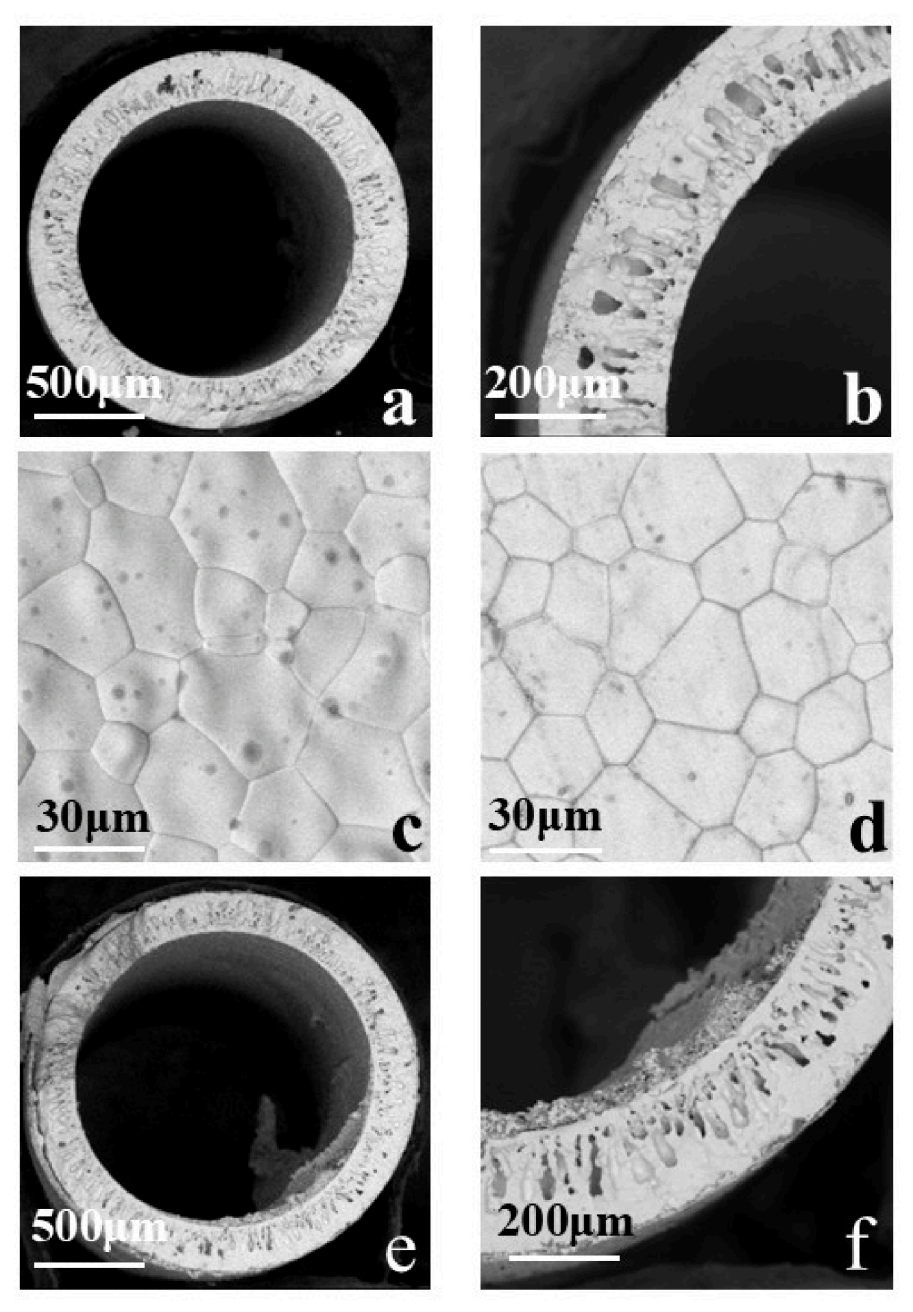
The BZCY hollow fiber membrane sintered at 1480 °C for 5 h: (**a**) cross-section; (**b**) the magnified cross-section; (**c**) inner surface and (**d**) outer surface; (**e**) cross-section after sintering the silver paste at 750 °C; (**f**) the magnified cross-section after sintering the silver paste.

The nitrogen permeance of the as-prepared hollow fiber membrane was measured to be 1.08 × 10^−9^ mol⋅m^−2^⋅s^−1^⋅Pa^−1^ at 0.35 MPa using a nitrogen testing device. This value is negligible compared with the hydrogen and oxygen permeation fluxes during the separation process, demonstrating that the membrane sintered at 1480 °C is dense. Figure 1 presents the scanning electron microscopy (SEM) images of the sintered BZCY ceramic hollow fiber membranes fabricated via phase inversion spinning. As can be seen from Figure 1a,b, the hollow fiber membranes exhibit a uniform tubular cross-section and well-defined asymmetric structure with a wall thickness of approximately 200 μm. The porosity of BZCY measured by the Archimedes method is 25%. As shown in Figure 1c,d, the surface exhibits smooth and pore-free morphology, with well-defined crystal cells and distinct grain boundaries. At room temperature, the gas tightness of the prepared hollow fiber membrane was tested under hydrogen and oxygen atmospheres, respectively. The results indicate that the membranes remained free of leaks after high-temperature sintering at 1480 °C. Figure 1e,f show the cross-section and the magnified cross-section of the membrane after being coated with silver paste. The porous silver paste layer was formed on the membrane surface after sintering at 750 °C, which is used for current collection. Meanwhile, as a common electrode material, silver also facilitates the catalytic dissociation of water vapor, hydrogen, and oxygen, thereby promoting surface electrochemical reactions.

**Figure 2:**
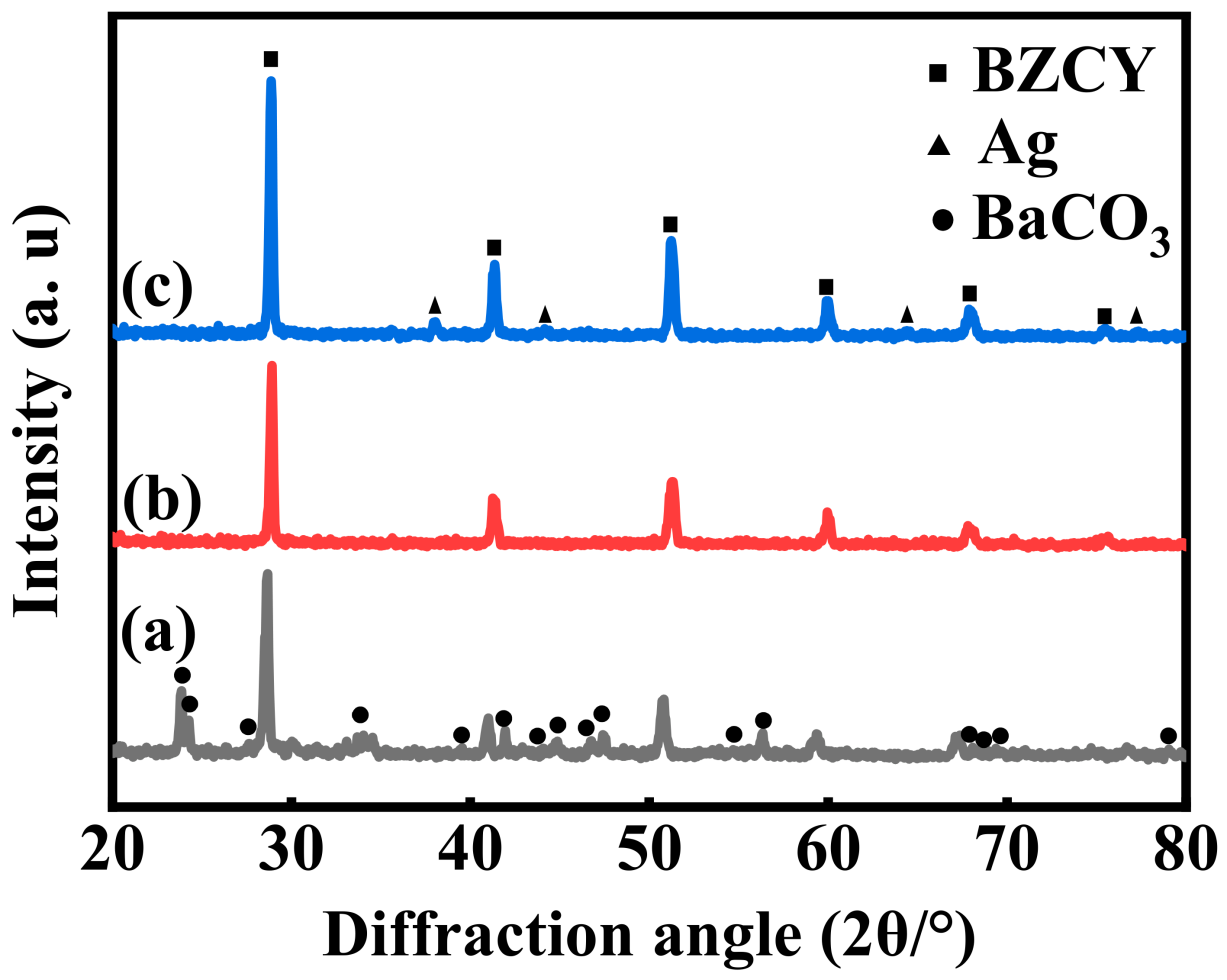
XRD diffraction images. (**a**) The BZCY powder after heat treatment at 1000 °C for 3 h; (**b**) BZCY hollow fiber sintered at 1480 °C; (**c**) BZCY hollow fiber membrane after sintering the silver paste at 750 °C.

Figure 2a presents the XRD patterns of BZCY powder calcined at 1000 °C for 3 h. The patterns indicate an incomplete formation of the perovskite phase. In contrast, the BZCY hollow fiber sintered at 1480 °C for 5 h exhibits the single-phase perovskite structure characteristic of BZCY, as confirmed in Figure 2b. The silver paste was applied to the membrane surface for current collection. After treatment at 750 °C, metallic silver formed on the membrane surface. XRD results shown in Figure 2c showed that no new compound phases appeared except for the additional peaks of metallic silver, proving that this treatment does not cause reaction between BZCY and silver, thus ensuring the integrity of BZCY.

### 2.2. Hydrogen and Oxygen Permeation Under Dry Atmosphere

**Figure 3:**
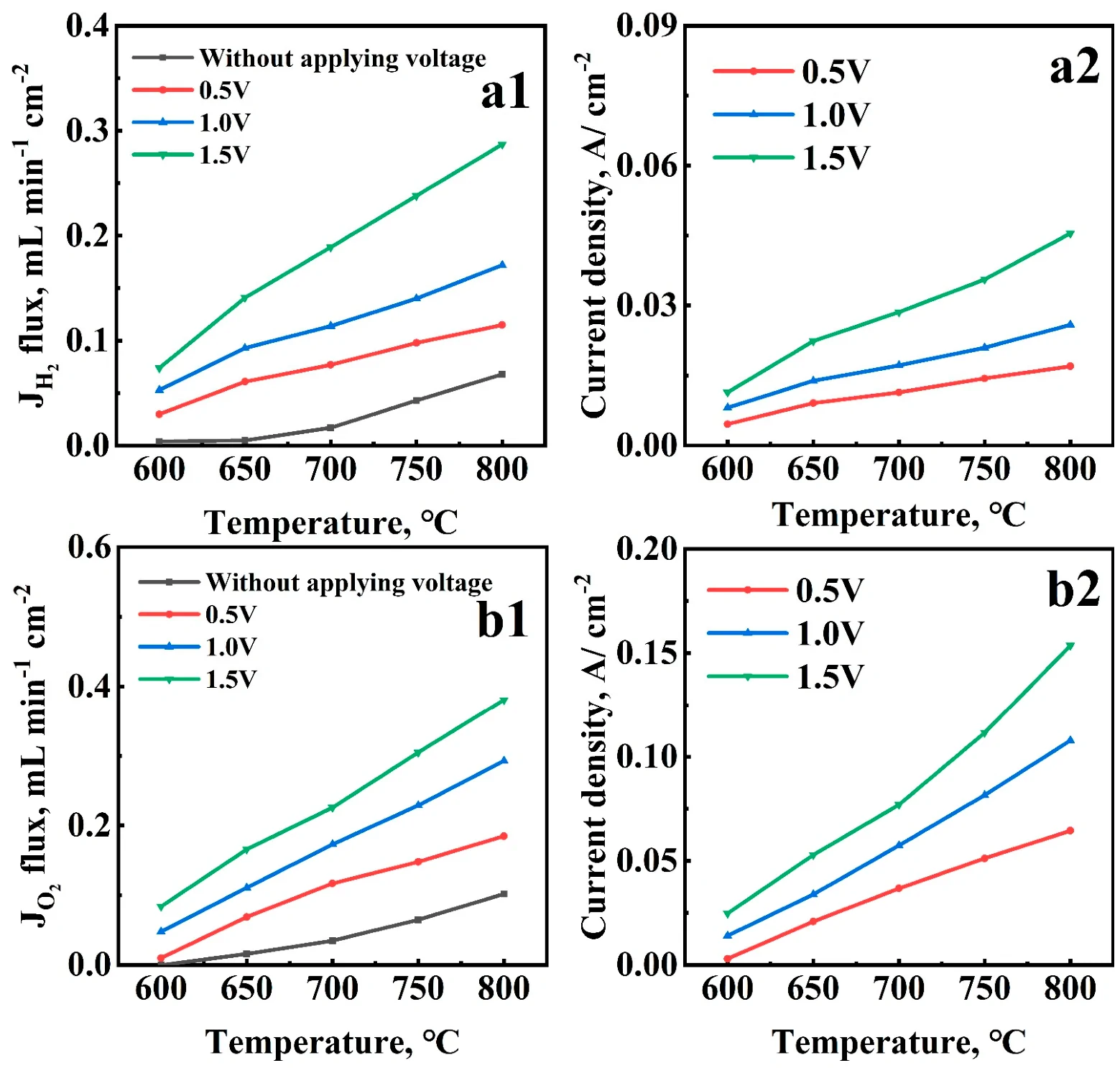
Hydrogen and oxygen permeation flux of BZCY HF as function of temperature and voltage in dry conditions (**a1**,**b1**); current density during hydrogen and oxygen permeation (**a2**,**b2**). (Hydrogen permeation test: cathode side: F_N_2__ = 100 mL min^−1^, anode side: F_40%H_2_−He_ = 30 mL min^−1^; oxygen permeation test: cathode side: F_Air_ = 100 mL min^−1^, anode side: F_He_ = 30 mL min^−1^).

Hydrogen and oxygen permeation through BZCY hollow fiber membranes was firstly tested under dry conditions in an electrically driven mode. The experimental results demonstrate that this membrane exhibits hydrogen and oxygen permeation properties as shown in Figure 3, which is primarily attributed to the oxygen ion and proton conductivity of BZCY. Figure 3(a1,b1) show that both H_2_ and O_2_ permeation fluxes increase progressively with rising temperature and applied voltage. For instance, at 1.5 V, raising the temperature from 600 to 800 °C enhances the hydrogen permeation flux by about 3 times, while the oxygen permeation flux increases by 3.5 times under the same conditions. As shown in Figure 3(a2,b2), the current density increases with the rise of temperature and voltage. The increasing trend of the current aligns with the rise in hydrogen and oxygen permeation, indicating that the current generation mainly relies on the transport of protons and oxygen ions. However, as evident from Figure 3, the BZCY membrane exhibits residual hydrogen and oxygen permeation at elevated operating temperatures (≥650 °C) without any applied voltage (non-electrically driven mode). This suggests the presence of minor electronic conductivity in the BZCY membrane material. By comparing the Faraday efficiency (i.e., the ratio of the actual hydrogen/oxygen permeation flux to its theoretical value), it can be known that at 600 °C and 0.5 V, the Faraday efficiency of the hydrogen permeation process is 98%, while that of oxygen is 97%. With the increase in voltage and temperature, electron conduction is more likely to occur in the oxygen permeation process. At 800 °C and 1.5 V, the Faraday efficiency of oxygen permeation is only 71%.

These findings confirm that elevated temperature and an applied electric field circuit can simultaneously promote both hydrogen and oxygen permeation. This outcome provides a baseline reference for further optimizing dual gas permeation performance with the introduction of water vapor.

**Figure 4:**
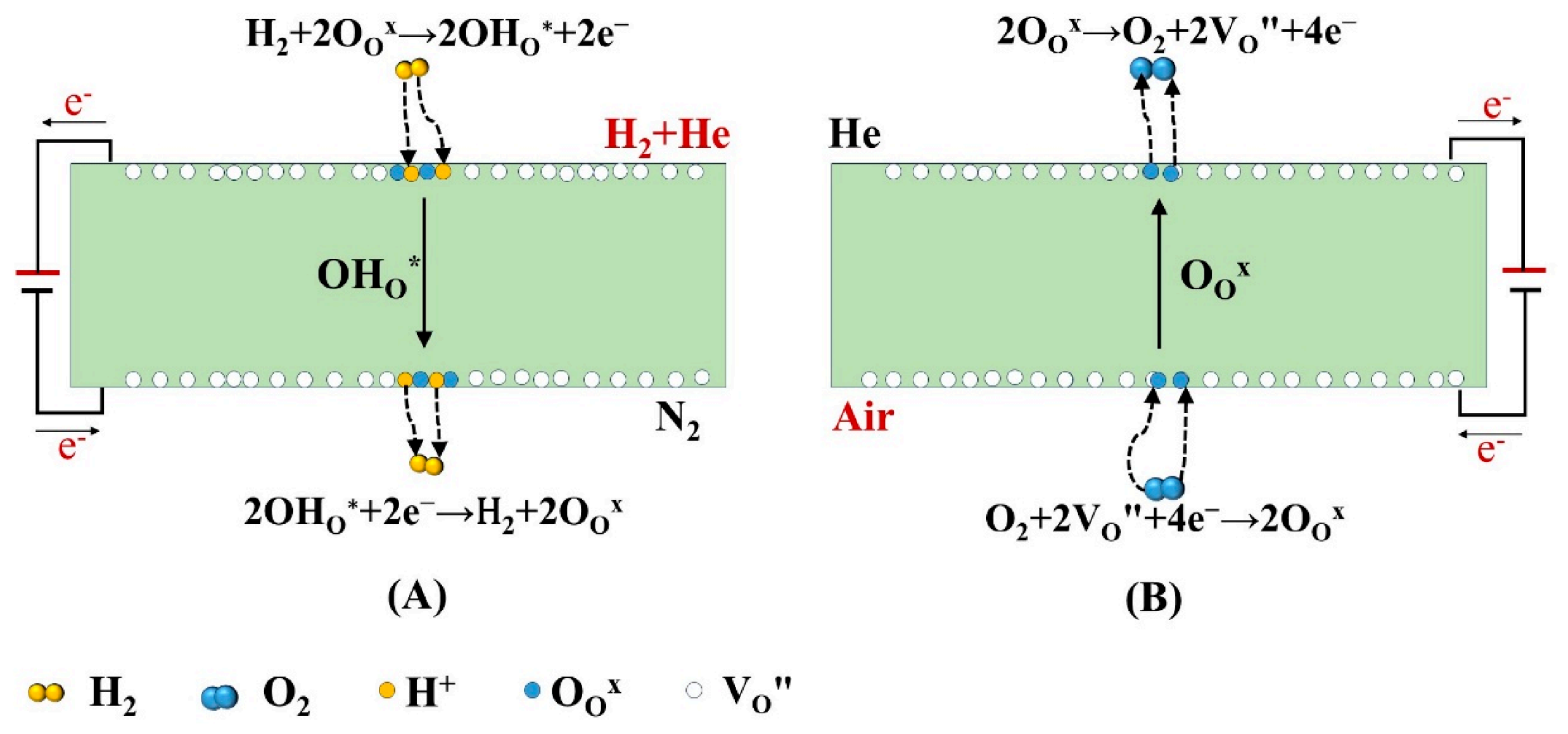
The permeation mechanism diagram of hydrogen (**A**) and oxygen (**B**) in BZCY HF membrane.

The permeation mechanisms of hydrogen and oxygen under dry atmosphere conditions can be explained in Figure 4, highlighting proton migration through lattice defects and oxygen vacancy-mediated diffusion pathways. As shown in Figure 4A, hydrogen permeation follows the Grotthuss mechanism [39,40]. The process initiates with gaseous H_2_ adsorption on the anode surface, where hydrogen molecules undergo electrochemical oxidation (H_2_ → 2H^+^ + 2e^−^) under the applied voltage. The generated protons (H^+^) then interact with lattice oxygen (O_O_^x^) in the membrane, forming quasi free proton defects (OHo*) that migrate through the oxygen lattice framework. At the cathode side, these protons recombine with electrons to form hydrogen (2H^+^ + 2e^−^ → H_2_), while simultaneously releasing lattice oxygen species, thereby completing the hydrogen permeation cycle.

The mechanism of oxygen permeation was depicted in Figure 4B, at the cathode side, gaseous oxygen adsorbs on the membrane surface and undergoes electrochemical reduction to oxygen ions (O_2_ + 4e^−^ → 2O^2−^), facilitated by oxygen vacancies and driven by the applied voltage [41]. The resulting O^2−^ ions occupy oxygen vacancy sites, forming mobile lattice oxygen species that migrate through the membrane under the influence of the electric potential gradient. At the anode side, these oxygen species are oxidized (2O^2−^ → O_2_ + 4e^−^), releasing electrons that complete the circuit while regenerating gaseous oxygen molecules, thereby achieving oxygen permeation through the membrane.

Notably, the present permeation mechanism is distinct from gas separation by water electrolysis. Protons for hydrogen permeation come from H_2_ molecules rather than decomposed water vapor, while oxygen ions for oxygen permeation are produced from gaseous O_2_ instead of steam. The applied external voltage merely boosts the ion migration driving force and does not induce water splitting reactions on electrodes.

Without applied voltages, the driving force for hydrogen and oxygen permeation relies solely on the partial pressure gradient across the membrane. Both the transport kinetics of protons/oxygen ions and the electronic conductivity within the membrane remain extremely limited, resulting in lower permeation fluxes. Both hydrogen and oxygen permeation rates are mainly controlled by the applied voltage and operating temperatures. Higher applied voltage could increase the driving force for ion diffusion, and higher temperatures could enhance the ions (protons and oxygen ions)’s conductivity in BZCY membrane materials.

### 2.3. Water Vapor Permeation Performance

**Figure 5:**
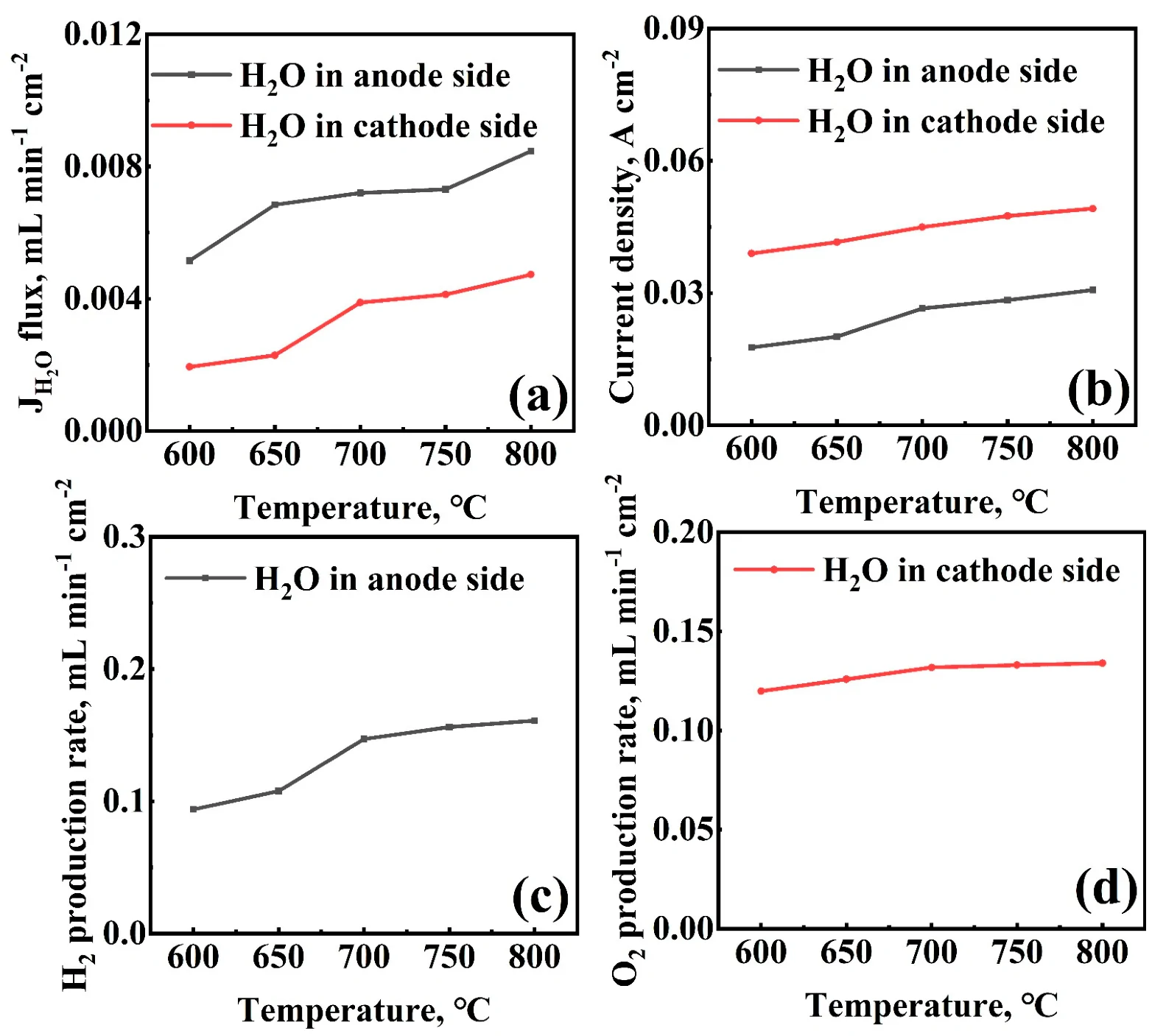
Under 1.5 V voltage, water vapor permeation performance and electrolysis as function of temperature. (**a**) Water vapor permeation flux on anode side and cathode side; (**b**) the current density of water vapor on anode and cathode side; (**c**) H_2_ permeation flux when water vapor is applied to the anode side; (**d**) O_2_ permeation flux when water vapor is applied to the anode side (feed side: F_N_2__ = 100 mL min^−1^, wet; sweep side: F_He_ = 30 mLmin^−1^).

The results presented in Figure 5 reveal that the BZCY membrane possesses weak water permeability. Figure 5a shows the water permeation flux when water vapor is introduced on the anode side or cathode side. It can be seen that the water permeation flux increases with rising temperature. Moreover, the water permeation flux is higher when water vapor is supplied to the anode side compared with the cathode side. Figure 5c,d present the permeation fluxes of hydrogen and oxygen formed by transmembrane transport, where these gases are produced via water vapor electrolysis with vapor supplied to the feed side. The results confirm that water vapor electrolysis occurs under an applied external voltage. The hydrogen produced by electrolysis when water vapor is fed to the anode side migrates toward the cathode side, generating the hydrogen permeation flux. By contrast, the oxygen generated by water vapor electrolysis at the cathode side migrates toward the anode side, forming an oxygen permeation flux. Comparison with Figure 5b shows that the variation trend of current density is highly consistent with that of hydrogen and oxygen permeation fluxes. Furthermore, the transport amount of hydrogen and oxygen is considerably larger than that of water vapor. Accordingly, the observed current response is mainly dominated by ion transport associated with hydrogen and oxygen permeation.

**Figure 6:**
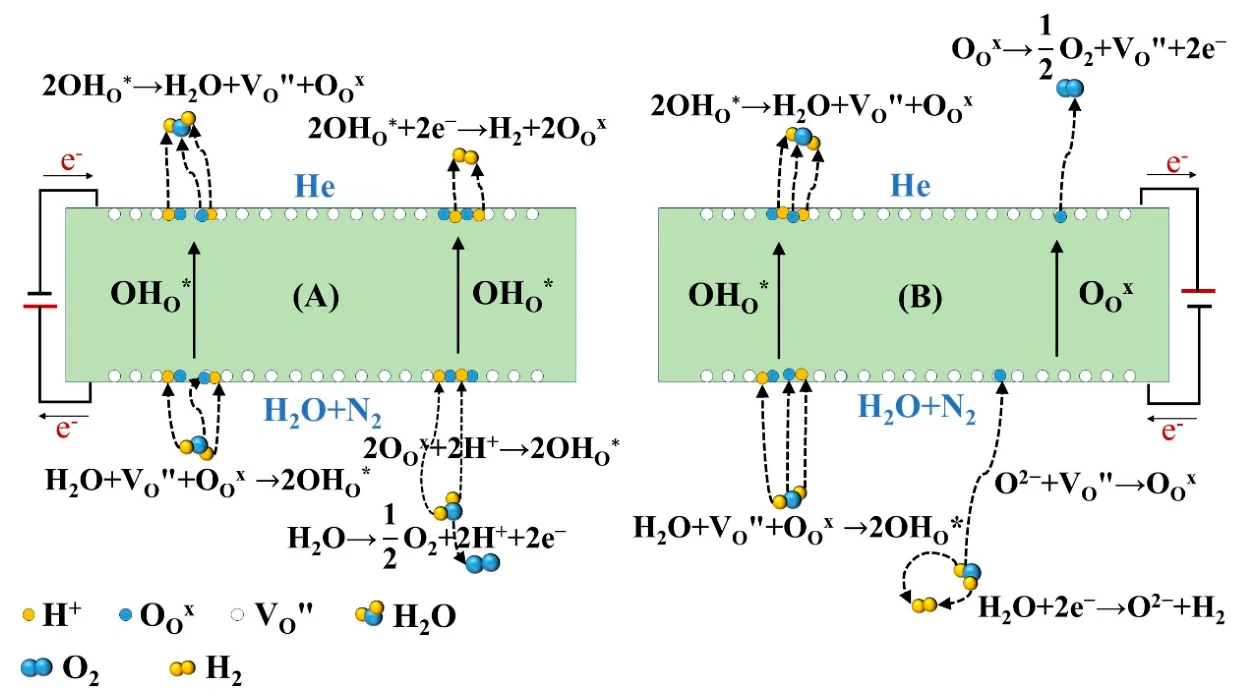
Schematic of water vapor permeation and electrolysis mechanisms when water vapor exists on the anode side (**A**) and cathode side (**B**) respectively.

Figure 6 illustrates the permeation mechanism of water vapor within the membrane. In humid environments, perovskite oxides demonstrate the characteristic hydration process wherein water vapor incorporates into oxygen vacancies through the Wagner reaction ($H_{2}O+{V_{O}}^{\prime \prime }+{O_{O}}^{X}\to {{2\mathrm{OH}}_{O}}^{*}$), generating protonic defects OH_O_* [35,42]. For example, when water vapor is on the anode side, similar to the hydrogen permeation process, quasi-free protons near oxygen ions can jump from one lattice to another through the Grotthuss mechanism. The water vapor recombines to form water vapor on the cathode side [34]. The applied positive voltage enhances OH_O_* migration due to the electrostatic force between the positively charged defects and the electric field. Conversely, on the cathode side, OH_O_* migration is hindered by the opposing electric field. Therefore, the permeation flux of water vapor applied on the anode side is higher than that on the cathode side.

The phenomenon presented in Figure 5c,d essentially constitutes a high-temperature solid oxide electrolysis cell (SOEC) [43]. As shown in Figure 6, water vapor is electrolyzed into H^+^ and O_2_ at the anode side. Driven by positive voltage, H^+^ can transfer through the membrane. Water vapor is electrolyzed into O^2−^ and H_2_ at the cathode side, due to the influence of negative voltage, and the O^2−^ on the cathode side migrates to the anode side. It should be noted that, under identical operating conditions, the theoretical amount of electrolytically generated hydrogen is twice that of oxygen.

### 2.4. Hydrogen Permeation Performance at Different Water Vapor Locations

**Figure 7:**
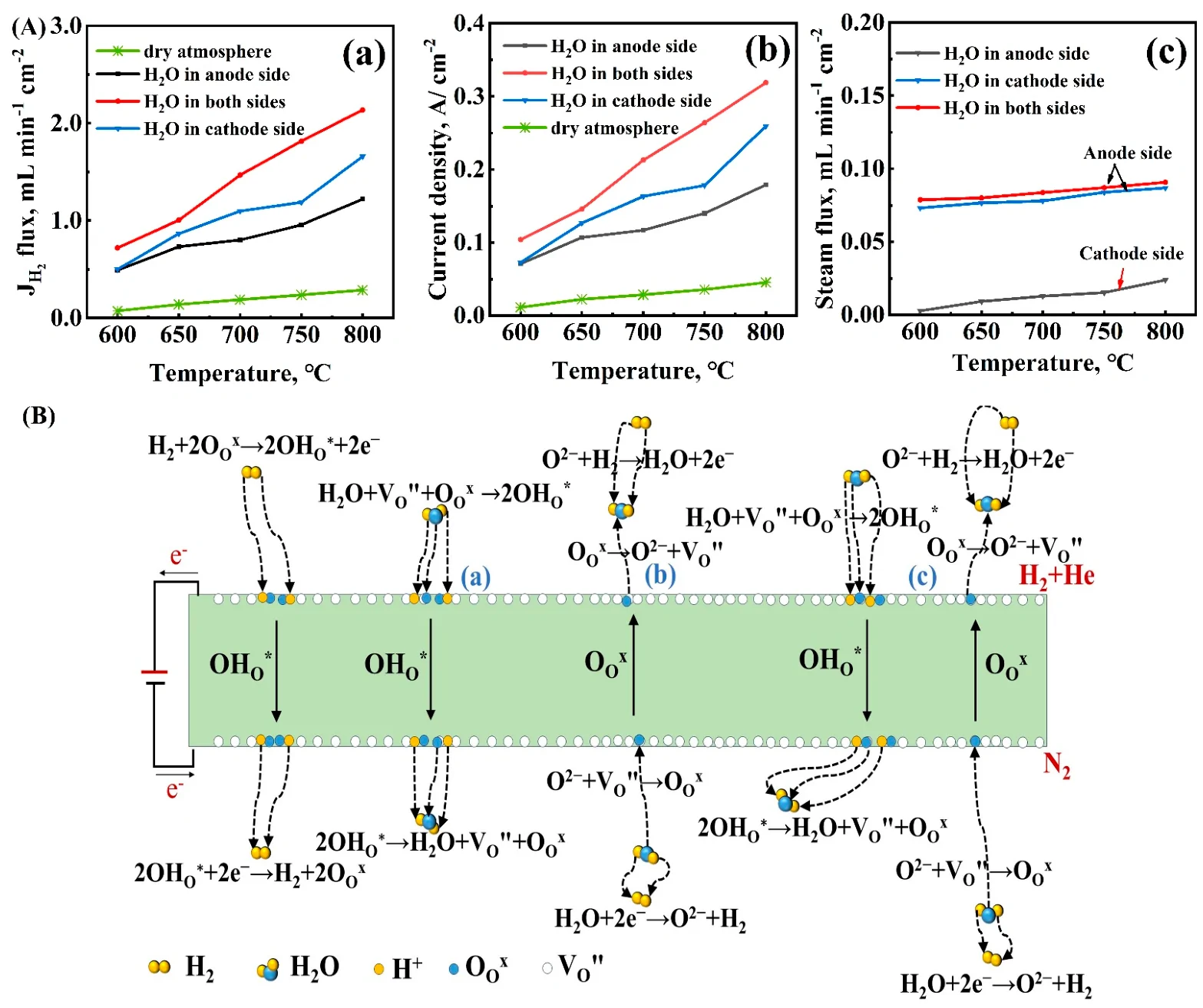
Under 1.5 V voltage, (**a**) hydrogen permeation flux; (**b**) the current density and (**c**) steam flux with temperature under different water vapor application positions are shown in (**A**); (**B**) is the mechanism diagram of the influence of different application positions of water vapor on hydrogen permeation performance; (**a**) water vapor is applied to the anode side; (**b**) water vapor is applied to the cathode side; (**c**) water vapor is applied to both sides. (Cathode side: F_N_2__ = 100 mL min^−1^; anode side: F_40%H_2_−He_ = 30 mL min^−1^).

As shown in Figure 7(Aa), the humid atmosphere can promote hydrogen permeation. The presence of water vapor facilitates proton conductivity due to the formation of OH_O_* from the reaction between the water vapor and oxygen vacancies as shown in Figure 7B. The increase in the current density also revealed the consistent phenomenon, as shown in Figure 7(Ab). Hence, all hydrogen flux can be enhanced irrespective of water vapor location. Combined with the water vapor permeation and electrolysis phenomena shown in Figure 5, when water vapor and hydrogen are fed on the same side, water vapor can generate OHo* during transmembrane transport, raising the proton concentration on the anode side and facilitating proton migration. Therefore, the enhancement in hydrogen flux is primarily attributed to the improved proton conductivity resulting from membrane hydration. The hydrogen permeation flux is higher when water is supplied to the cathode side compared with the anode side. When water vapor is present at the cathode, its electrolysis generates oxygen ions that migrate toward the anode. This results in substantial lattice oxygen accumulation at the anode, which establishes efficient transport pathways for proton conduction ($H^{+}+{O_{O}}^{x}\to {{\mathrm{OH}}_{O}}^{*}$). Consequently, the hydrogen permeation flux is significantly enhanced. Additionally, the transported oxygen reacts with hydrogen at the anode, producing water vapor, serving as the origin of the steam observed in Figure 7(Ac). The relatively low steam flux suggests that the amount of oxygen transported is limited. Consequently, the hydrogen generated via electrolysis is negligible when compared with enhanced hydrogen permeation. This supports the conclusion that the rise in water permeation is not attributable to water electrolysis. When water vapor is present on both sides of the membrane, the membrane hydration is enhanced. Coupled with the transport of oxygen ions (water electrolysis on the cathode) to the anode that creates abundant lattice oxygen channels, this configuration yields optimal hydrogen permeation performance. For example, the presence of water vapor on both sides significantly enhances hydrogen permeation flux, increasing by about 6 times at 800 °C (1.5 V) relative to dry atmospheric conditions.

**Figure 8:**
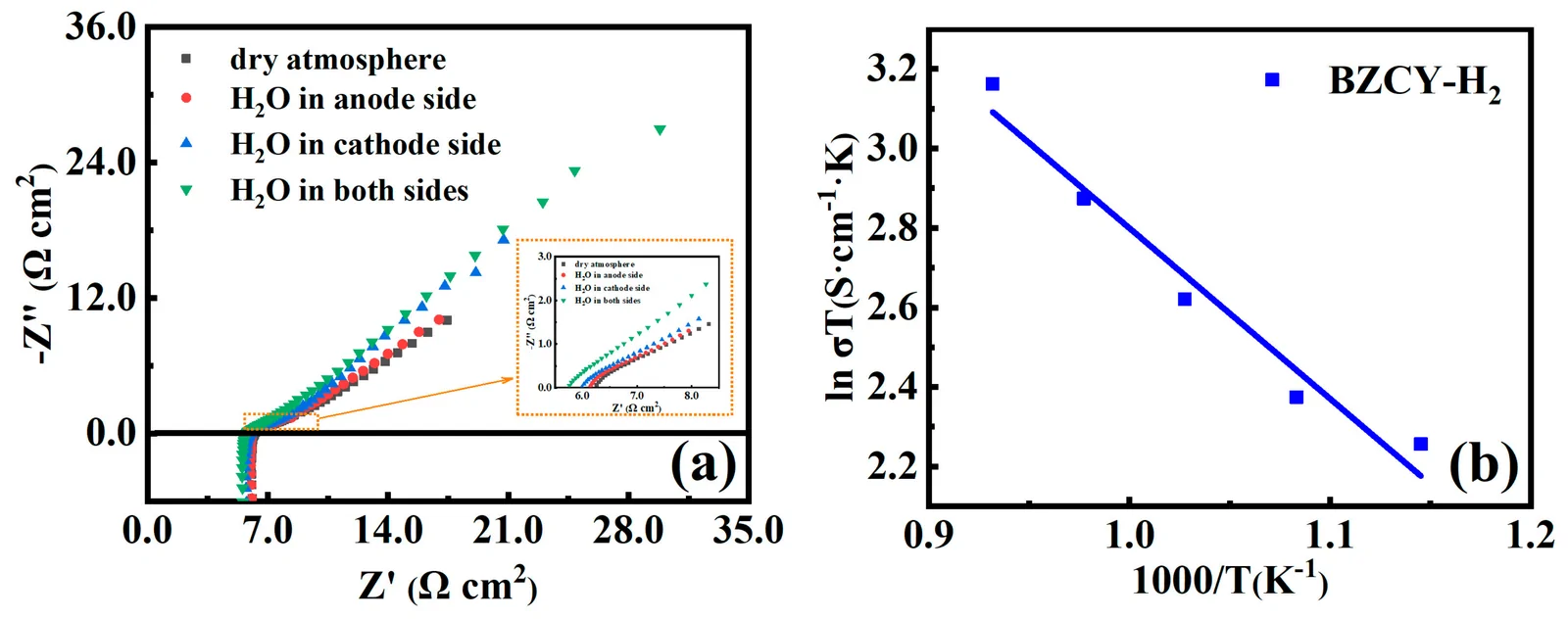
(**a**) Impedance spectra under 0.5 V, 600 °C in H_2_ atmosphere with water vapor introduced at different positions; (**b**) temperature dependence of conductivity of the BZCY hollow fiber membrane in hydrogen atmosphere.

To further verify the above conclusions, EIS analysis was also conducted at 0.5 V to investigate the effect of water vapor introduced at different positions on hydrogen permeation. As can be seen in Figure 8a, the smallest resistance is observed when water vapor is supplied on both sides simultaneously, which is consistent with the phenomenon observed in Figure 7(Aa). In Figure S1 of the Supplementary Materials, the impedance spectra covering the range from high to low frequencies are displayed in detail. Figure 8b shows the variation of conductivity with temperature under a hydrogen atmosphere, and the activation energy calculated from the Arrhenius equation is 36 kJ·mol^−1^.

### 2.5. Oxygen Permeation Performance at Different Water Vapor Locations

**Figure 9:**
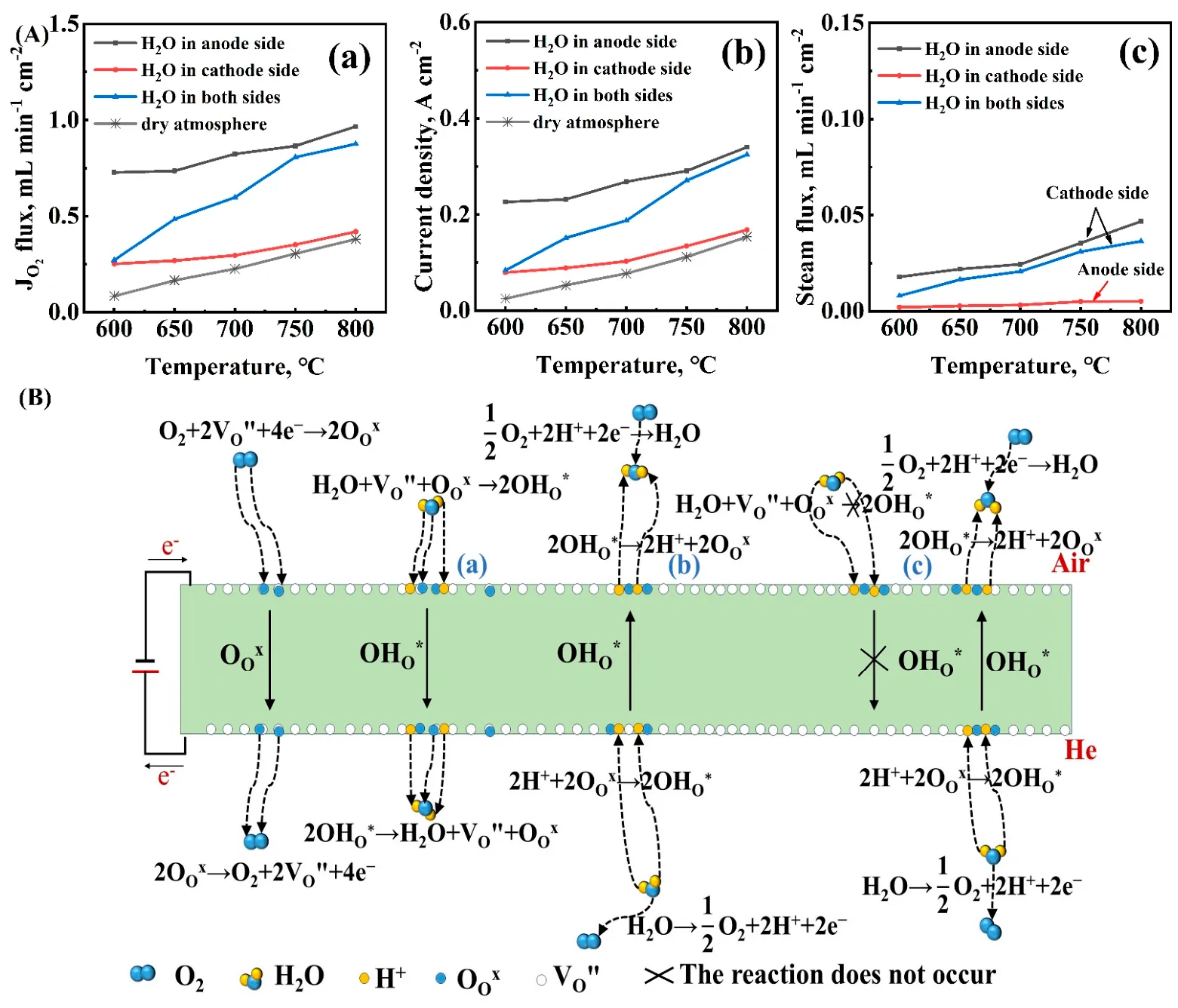
Under 1.5 V voltage, (**a**) oxygen permeation flux; (**b**) the current density; (**c**) steam flux with temperature under different water vapor application positions are shown in (**A**). (**B**) is the mechanism diagram of the influence of different application positions of water vapor on oxygen permeation performance; (**a**) water vapor is applied to the cathode side; (**b**) water vapor is applied to the anode side; (**c**) water vapor is applied to the both sides. (Cathode side: F_Air_ = 100 mL min^−1^; anode side: F_He_ = 30 mLmin^−1^).

Figure 9A presents the investigation of temperature-dependent oxygen permeation under different water vapor configurations. As shown in Figure 9(Aa), the presence of water vapor also contributes to the enhancement of the oxygen permeation flux. At 1.5 V and 800 °C, the presence of water vapor on the anode side can increase the oxygen flux by approximately 1.5 times relative to that under a dry atmosphere.

The mechanism underlying the enhanced oxygen permeation performance under a humid atmosphere is illustrated in Figure 9B. Compared with dry conditions, cathode-side humidification demonstrates the modest enhancement in oxygen permeation. This phenomenon can be attributed to the formation of OH_O_* intermediates ($H_{2}O+{\mathrm{Vo}}^{\prime \prime }+{O_{O}}^{x}\to {{2\mathrm{OH}}_{O}}^{*}$) in the presence of trace water vapor, as shown in Figure 9(Ba), which reduces the activation energy of surface reactions and accelerates oxygen adsorption/desorption kinetics, thereby improving permeation performance [44].

The oxygen permeation flux is most significantly enhanced when the anode side is humidified. Notably, the observed increase in flux is seldom attributed to oxygen generated by water electrolysis. Under such an atmosphere, as illustrated in Figure 9(Bb), protons generated via water electrolysis at the anode can be subsequently transported to the cathode and react with air to form steam. Figure 9(Ac) confirms minimal steam generation from proton/air reactions at the cathode. Quantitative analysis reveals that electrolytically produced oxygen constitutes less than 10% of the total flux enhancement. Rather, the improvement primarily stems from proton counter permeation, which likely enhances the thermodynamic driving force for oxygen ion transport. When water vapor is applied to the cathode side, an increase in current density can be observed in Figure 9(Ab) compared with dry atmosphere. When water vapor is introduced to both sides of the membrane, permeation improvement exhibits intermediate values. Figure 9(Ac) shows reduced cathode steam production (indicating diminished proton flux) compared with anode-only humidification, explaining the attenuated permeation enhancement. In summary, the proton counter permeation directly correlates with oxygen permeation performance.

**Figure 10:**
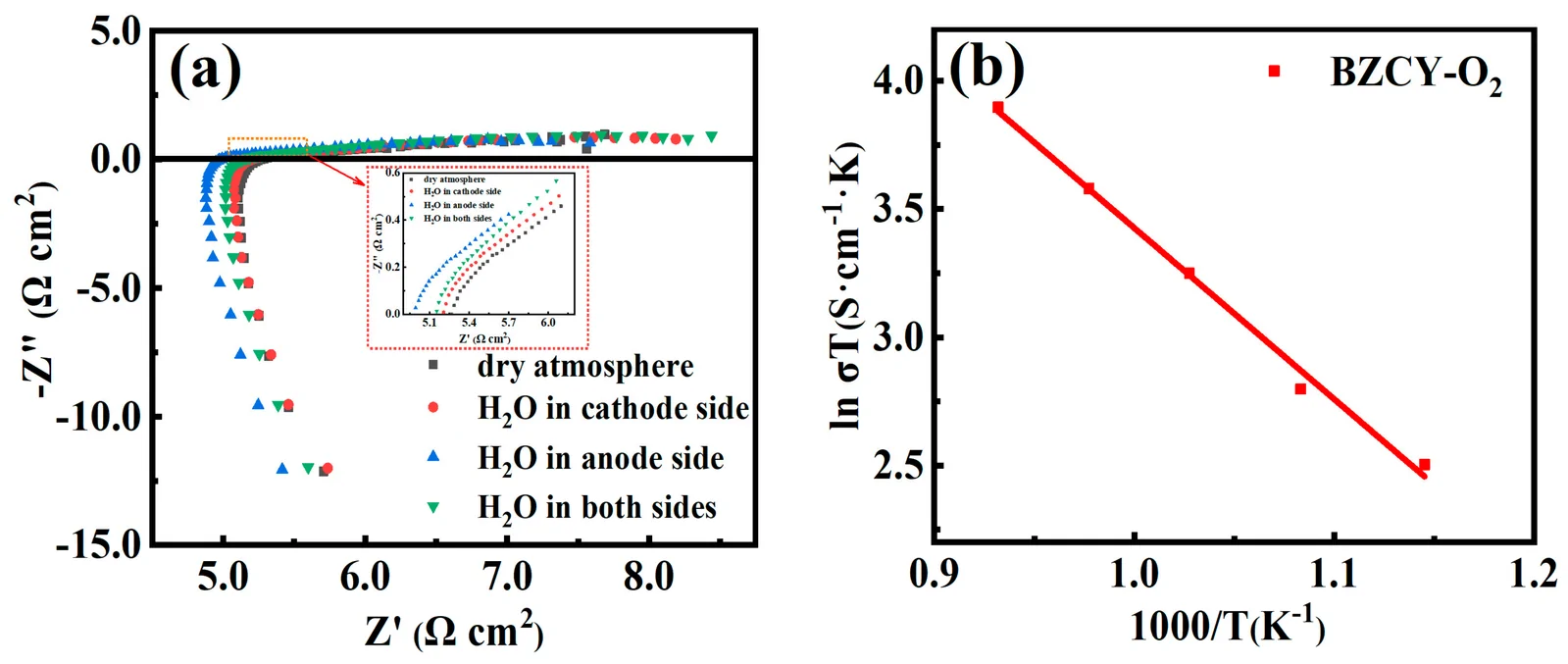
(**a**) Impedance spectra under 0.5 V, 600 °C in O_2_ atmosphere with water vapor introduced at different positions; (**b**) temperature dependence of conductivity of the BZCY hollow fiber membrane in oxygen atmosphere.

According to the impedance spectra shown in Figure 10a, measured at 0.5 V and 600 °C under oxygen atmosphere with water vapor introduced at different positions, the lowest impedance value is observed when water vapor is supplied on the anode side. This indicates that introducing water vapor on the anode side is favorable for the permeation and diffusion of oxygen, which is consistent with the results shown in Figure 9(Aa). Figure S2 in the Supplementary Materials presents the full impedance spectra ranging from high to low frequencies under different water vapor feeding positions in detail. In addition, Figure 10b displays the temperature dependence of conductivity for BZCY under oxygen atmosphere. The activation energy calculated from the plot is 55 kJ·mol^−1^.

Table 1 compares the hydrogen and oxygen permeation fluxes reported in the literature. Analysis on the permeation performance of different materials indicates that the application of external voltage can greatly enhance the membrane permeability, with a particularly prominent improvement in oxygen permeation flux. Without external voltage and steam supply, such membranes can only achieve excellent oxygen separation performance at temperatures above 900 °C.

Most existing studies have not simultaneously investigated hydrogen and oxygen separation behaviors under external voltage, and the relevant research under steam atmosphere is even less common. The experimental results reveal that steam can not only significantly increase the permeation fluxes of hydrogen and oxygen but can also undergo electrolysis and participate in the permeation process. The findings provide an important reference for the design of membrane reactors dedicated to methane aromatization. Previous studies have verified that double-layer BZCY hollow fiber membranes can effectively boost the performance of methane aromatization in the presence of steam [21]. In follow-up work, nickel-based current collecting layers and catalytic layers will be fabricated on the surface of BZCY electrolyte membranes to further improve the separation performance of membrane tubes. Meanwhile, the current collecting layers can promote steam electrolysis, thereby optimizing the overall performance of the membrane reactor during the aromatization reaction.

## 3. Experimental

### 3.1. Fabrication of BZCY Hollow Fiber Membranes

BaZr_0.2_Ce_0.7_Y_0.1_O_3−δ_ (BZCY) hollow fiber (HF) membranes were fabricated via a combined phase inversion and sintering process. BaZr_0.2_Ce_0.7_Y_0.1_O_3−δ_ (BZCY) perovskite powder was prepared by a high-temperature solid-state reaction method. BaCO_3_, CeO_2_, ZrO_2_, and Y_2_O_3_ were added into an agate jar sequentially according to the molar ratio required for BZCY, and ball mill with ethanol as dispersant for 10 h. Subsequently, the dried powder was calcined at 1000 °C for 3 h and sieved to obtain a uniform powder with a 200 mesh size.

Using N-methyl-pyrrolidone (NMP) (AR grade, >99.8%, Kermel Chemical, Tianjin, China) as the solvent and polysulfone (PSF) (Udel 3500, Solvay, Alpharetta, GA, USA) as the binder, after uniform stirring, the BZCY powder and sintering aid (1 wt% NiO) were added and continuously stirred for 48 h. Then, a single-layer customized spinneret with an orifice diameter/inner diameter of 3.0/1.5 mm was used to prepare the hollow fiber membrane precursor. The fully cured precursor was cut into appropriate lengths and dried out. The dried hollow fiber precursors were sintered at 1480 °C under a non-flowing air atmosphere with a heating rate of 3 °C min^−1^. Silver paste (Sino-Platinum Metals Co., Ltd., Kunming China) was coated on both the inner and outer sides of the sintered BZCY hollow fiber membrane, and silver wires were wound around it. And then the membranes with silver pastes coated were sintered in the tube furnace at 750 °C.

### 3.2. Characterization

The micromorphology of the BZCY hollow fiber membranes was characterized using a scanning electron microscope (SEM, Hitachi S4800, Tokyo, Japan). Before scanning the samples, the surface of the prepared samples was sputtered with gold. The crystalline structure of the BZCY membranes was analyzed by using an X-ray diffractometer (XRD, D8 Advance, Bruker, Karlsruhe, Germany). Before XRD testing, the BZCY membranes were ground into powder form. The XRD patterns were collected over a 2θ range of 20° to 80° using Cu-Kα radiation (λ = 0.15418 nm). The data were acquired with a sampling interval of 0.02° and a scanning speed of 2° min^−1^, using an X-ray tube operated at 40 kV and 40 mA.

The gas tightness of the as-prepared hollow fiber membranes was tested using a nitrogen permeation apparatus at room temperature. The detailed test procedure has been described elsewhere [54]. The hollow fibers with sealed ends were bonded to a sample holder with epoxy resin, and the assembly was then placed into a stainless-steel cylinder. The nitrogen permeance was calculated according to the permeate flow rate measured by a bubble flow meter. This flow meter has a minimum detectable volume of 0.05 mL. The calculation formula is: $P=\frac{\mathrm{PaV}/\mathrm{RT}}{{\mathrm{Am}}^{*}(p-\mathrm{Pa})}$, where P is the gas permeance of the hollow fiber membrane, mol⋅m^−2^⋅s^−1^⋅Pa^−1^; V represents the permeate gas flow rate, m^3^⋅s^−1^; R denotes the universal gas constant, which is 8.314 J⋅mol^−1^⋅K^−1^; T is the measurement temperature, K; Pa stands for the atmospheric pressure, Pa; p refers to the permeation pressure, Pa.

### 3.3. Hydrogen and Oxygen Permeation Measurement

**Figure 11:**
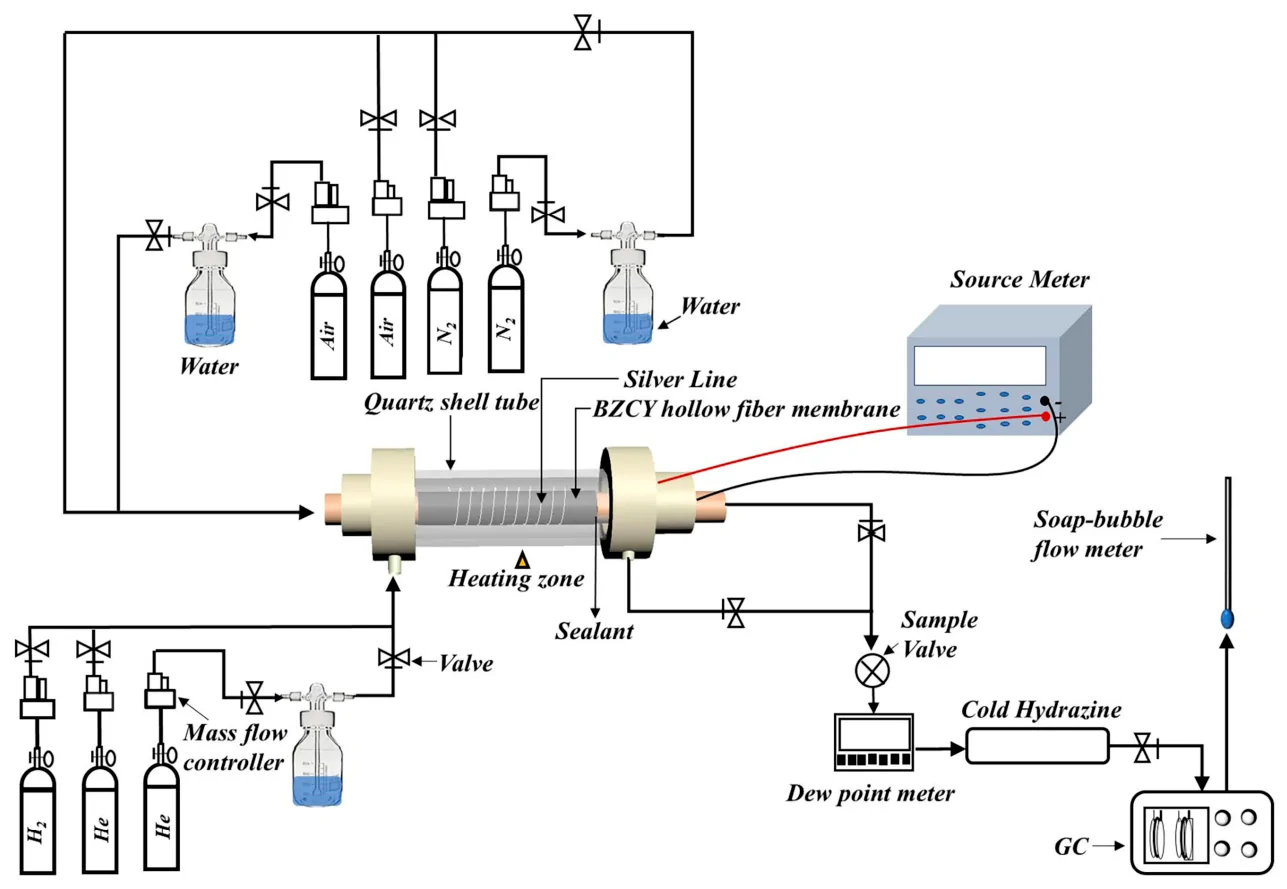
Experimental setup for BZCY hollow fiber membrane reactor test.

A self-made high-temperature hollow fiber membrane reactor was employed to investigate the effect of water vapor. The experimental setup is illustrated in Figure 11. The reactor was placed in an open-type tube furnace with a heating zone length of 5 cm. All membranes were longer than 20 cm, and high-temperature adhesive was used to seal the contact areas between both sides of the membrane and the quartz tube. For the oxygen permeation performance test, compressed air as the gas source was fed into the tube side and high-purity helium as the sweep gas was fed into the shell side. For the hydrogen permeation performance test, high-purity helium gas as the dilution gas to dilute the hydrogen concentration to 40% was fed into the shell side, and high-purity nitrogen gas as the sweep gas was fed into the tube side. The digital source instrument (Keithley 2440, Tektronix, Beaverton, OR, USA) was used to apply external voltage to the tube and shell sides of the hollow fiber membrane.

In order to test the performance of water vapor, water vapor was introduced into the membrane reactor by bubbling nitrogen gas through customized water bottles, and dry high-purity nitrogen gas was used as the diluent gas at the same time. The performance test of water vapor on the BZCY membrane was carried out by switching the control valves on the tube side and shell side to control the different transport positions of water vapor. During the permeation test, the dew point meters (DP900, Shanghai Toly Instruments Co., Ltd., Shanghai, China) were used to record the water vapor changes inside and outside the membrane in real time. The outlet gases on the tube and shell sides were analyzed using GC (Agilent 7890B, Agilent Technologies, Santa Clara, CA, USA) equipped with a 5 Å molecular sieve column (10 feet × 1 inch), which was equipped with FID and TCD detectors and used high-purity helium and argon as carrier gases. And the outlet gas flow rate was measured using a soap bubble flowmeter. The calculation formulas related to the flux of water vapor, hydrogen and oxygen are detailed in the Supplementary Materials.

## 4. Conclusions

This study systematically analyzes the permeation behaviors of steam, hydrogen and oxygen in electrochemical membrane reactors within the temperature range of 600–800 °C. The results reveal that steam addition remarkably improves both hydrogen and oxygen permeation fluxes. At the typical aromatization temperature of 700 °C and an applied voltage of 1.5 V, the hydrogen permeation flux increases to 1.469 mL⋅min^−1^⋅cm^−2^, in sharp contrast to 0.189 mL⋅min^−1^⋅cm^−2^ under a dry atmosphere. Such a remarkable improvement is beneficial for in situ hydrogen extraction under low hydrogen partial pressure during aromatization. Meanwhile, the oxygen permeation flux reaches 0.824 mL⋅min^−1^⋅cm^−2^ at 700 °C with steam, while only 0.226 mL⋅min^−1^⋅cm^−2^ is obtained under dry conditions. The enhanced oxygen flux enables a continuous oxygen supply from the permeate side to the reaction chamber for mitigating coke formation. The above results establish a solid foundation for the fabrication of electrochemical catalytic membrane reactors for methane dehydroaromatization.

## Figures and Tables

**Table 1:** Comparison of hydrogen and oxygen permeation fluxes of typical proton/oxygen ion conducting membranes at 600–800 °C.

Ref	Material	H_2_/O_2_ Permeation Flux	Testing Atmosphere and Temperatures
This study	BaZr_0.2_Ce_0.7_Y_0.1_O_3−δ_	H_2_: 1.469 mL min^−1^ cm^−2^; O_2_: 0.824 mL min^−1^ cm^−2^	40% H_2_,700 °C, 1.5 V, steam on both sides Air, 700 °C, 1.5 V, steam on the sweep side
[29]	BaCe_0.70_Fe_0.10_Sc_0.20_O_3−δ_	H_2_: ~0.04 mL min^−1^ cm^−2^; O_2_: ~0 mL min^−1^ cm^−2^;	83.3 vol%H_2_, 700 °C, steam on the feed side; Air, 700 °C, steam on the feed side
[45]	Ni-BaZr_0.1_Ce_0.7_Y_0.2_O_3−δ_/BaZr_0.1_Ce_0.7_Y_0.2_O_3−δ_/Ni-BaZr_0.1_Ce_0.7_Y_0.2_O_3−δ_	H_2_: 15.8 mL min^−1^ cm^−2^	50% H_2_, 500 °C, 2.0 V, steam on both sides
[46]	BaCe_0.7_Zr_0.1_Y_0.2_O_3−δ_/Ni-BaCe_0.7_Zr_0.1_Y_0.2_O_3−δ_	H_2_: ~1.8 mL min^−1^ cm^−2^	13% H_2_, 700 °C,2 V, steam on the feed side
[47]	BZF10	H_2_: ~0.47 mL min^−1^ cm^−2^	10% H_2_, 700 °C, steam on the feed side
[48]	Pd|BCFZY|Pd	H_2_: 0.213 mL min^−1^ cm^−2^	10% H_2_, 650 °C, dry atmosphere
[49]	BaCe_0.65_Zr_0.20_Y_0.15_O_3−δ_-Ce_0.85_M_0.15_O_2−δ_	H_2_: 0.27 mL min^−1^ cm^−2^	50% H_2_, 755 °C, steam on both sides
[50]	70 vol.% GDC + 30 vol.% LSCF/SCFN	O2: ~3.5 mL min^−1^ cm^−2^	Air, 800 °C, dry atmosphere
[51]	BSCF	O2: ~2.5 mL min^−1^ cm^−2^	Air, 700 °C, steam on the sweep side
[52]	Ce_0.8_Sm_0.2_O_1.9_-La_0.8_Sr_0.2_Cr_0.5_Fe_0.5_O_3−δ_	O2: 0.82 mL min^−1^ cm^−2^	Air, O_2_, 700 °C, dry atmosphere
[53]	Ba_0.975_La_0.025_Fe_1−x_Cu_x_O_3−δ_	O2: ~0.9 mL min^−1^ cm^−2^	Air, 800 °C, dry atmosphere

## Data Availability

The original contributions presented in this study are included in the article/Supplementary Material. Further inquiries can be directed to the corresponding authors.

## Supplementary Materials

The following supporting information can be downloaded at: https://www.mdpi.com/article/10.3390/membranes16080260/s1, Figure S1: Impedance spectra measured under 0.5 V at 600 °C in a H_2_ atmosphere with water vapor supplied at different positions, recorded from high to low frequency. (a) Dry atmosphere; (b) water vapor is applied to the anode side; (c) water vapor is applied to the cathode side; (d) water vapor is applied to both sides, Figure S2: Impedance spectra measured under 0.5 V at 600 °C in an O_2_ atmosphere with water vapor supplied at different positions, recorded from high to low frequency. (a) Dry atmosphere; (b) water vapor is applied to the cathode side; (c) water vapor is applied to both sides; (d) water vapor is applied to the anode side, Table S1: The gas components analyzed by GC on the permeate side at room temperature.
