# Supplementary material for: Promotion Effect of Steam on Hydrogen and Oxygen Separation in Electrochemical Membrane Reactor: Investigation Under the Aromatization Reaction Temperature Window
Source: Membranes (Basel). 2026 Jul 31;16(8):260. doi: 10.3390/membranes16080260 (PMC13515430; doi:10.3390/membranes16080260)
Supplement: Supplementary file 1 [file membranes-16-00260-s001.zip › membranes-4399671-supplementary.pdf]

membranes

MDPI

Supplementary Materials

Promotion Effect of Steam on Hydrogen and Oxygen Separation in Electrochemical Membrane Reactor: Investigation Under the Aromatization Reaction Temperature Window

Lihui Wang [1], Shao Zhang [1], Mingming Wang [1,2,*], Zhigang Wang [1,2,*] and Xiaoyao Tan [1,*]

[1] State Key Laboratory of Advanced Separation Membrane Materials, Department of Chemical Engineering, Tiangong University, Tianjin 300387, China; 2020020045@tiangong.edu.cn (L.W.); 2120020043@tiangong.edu.cn (S.Z.)
[2] Hebei Industrial Technology Research Institute of Membranes, Cangzhou Institute of Tiangong University, Cangzhou 061000, China
* Correspondence: wangmingming@tiangong.edu.cn (M.W.); wangzhigang@tiangong.edu.cn (Z.W.); tanxiaoyao@tiangong.edu.cn (X.T.)

1. The calculation formulas of permeation flux

Membrane area calculation formula, cm^{-2}:

$$\mathrm{Am} = \frac{\pi(D\text{-}d)l}{\ln(\frac{D}{d})} \quad \text{(S1)}$$

where L is the length of the membrane heating zone, cm; D and d are the inner and outer diameters of the membrane, cm.

$$\text{Hydrogen permeation flux: } J_{H_2}=\frac{\left(x_{H_2}-\frac{12}{18}x\mathrm{He}\right)\%\times \mathrm{Fout}}{A_m} \quad \text{(S2)}$$

$$\text{Oxygen permeation flux: } J_{O_2}=\frac{\left(x_{O_2}-\frac{21}{79}x\mathrm{N}_2\right)\%\times \mathrm{Fout}}{A_m} \quad \text{(S3)}$$

where x_i represents the gas content at the outlet of the permeation side, %; F_{out} denotes the gas flow rate at the outlet of the permeation side, mL/min; Am is the membrane area, cm^2.

$$\text{Water vapor permeation flux: } J_{H_2O}=\frac{y_{H_2O}\times \mathrm{Fout}}{A_m\times 10\text{\^{}}6} \quad \text{(S4)}$$

where y_{H2O} is the content of water vapor at different sides, ppm; F_{out} is the outlet gas flow rate of the sweep side, mL/min; Am is the membrane area, cm^{-2}.

Current density=I/Am, A cm^{-2}

Where I is the current value displayed by the digital source meter, A.

2. The conductivity and impedance characterization

The conductivity of the hollow fiber membranes was measured using a four-probe DC method. Prior to the test, silver wires were wrapped around both ends of the sample and fixed with silver paste. The sample was then connected to a Gamry potentiostat, and the corresponding currents were recorded at various applied voltages. The conductivity was calculated using the following equation.

$$\sigma = \frac{L}{R \times A} \quad (S5)$$

where σ represents the electrical conductivity of the sample, S cm^{-1}; L is the length of the sample, cm; A is the cross-sectional area of the measured sample ($A=\pi/4(d_o^2-d_i^2)$; and R is the electrical resistance of the sample obtained from the linear fitting of the measured I–V data, Ω. Impedance spectroscopy measurements were also performed using a Gamry electrochemical workstation.

The activation energies under dry hydrogen and air atmospheres were calculated using the Arrhenius equation: $\delta=(A/T)\ exp\ (-Ea\ /KT)$ (where *A*, *K*, and *T* are pre-exponential factor, Boltzmann constant and absolute temperature, respectively), and the activation energy for electrical conduction (*Ea*) was determined.

3. The porosity measurement

The porosity of the as-prepared hollow fiber membranes was measured via the Archimedes principle. Samples of appropriate size were selected and weighed as m0. The samples were then immersed in boiling deionized water for 2 h to fully infiltrate the pores. After saturation, the samples were suspended and weighed as m1. Finally, the samples were taken out, surface moisture was removed, and the samples were weighed again as m2. The porosity of the hollow fiber membranes was calculated using the following equation:

$$\xi = \frac{m2 - m0}{m2 - m1} \quad (S6)$$

Table S1. The gas components analyzed by GC on the permeate side at room temperature.

Gas	H_2	He	O_2	N_2
Hydrogen permeation test, %	0.02	0.017		99.96
Oxygen permeation test, %		100	0	0

4. The measurement frequency for impedance spectra

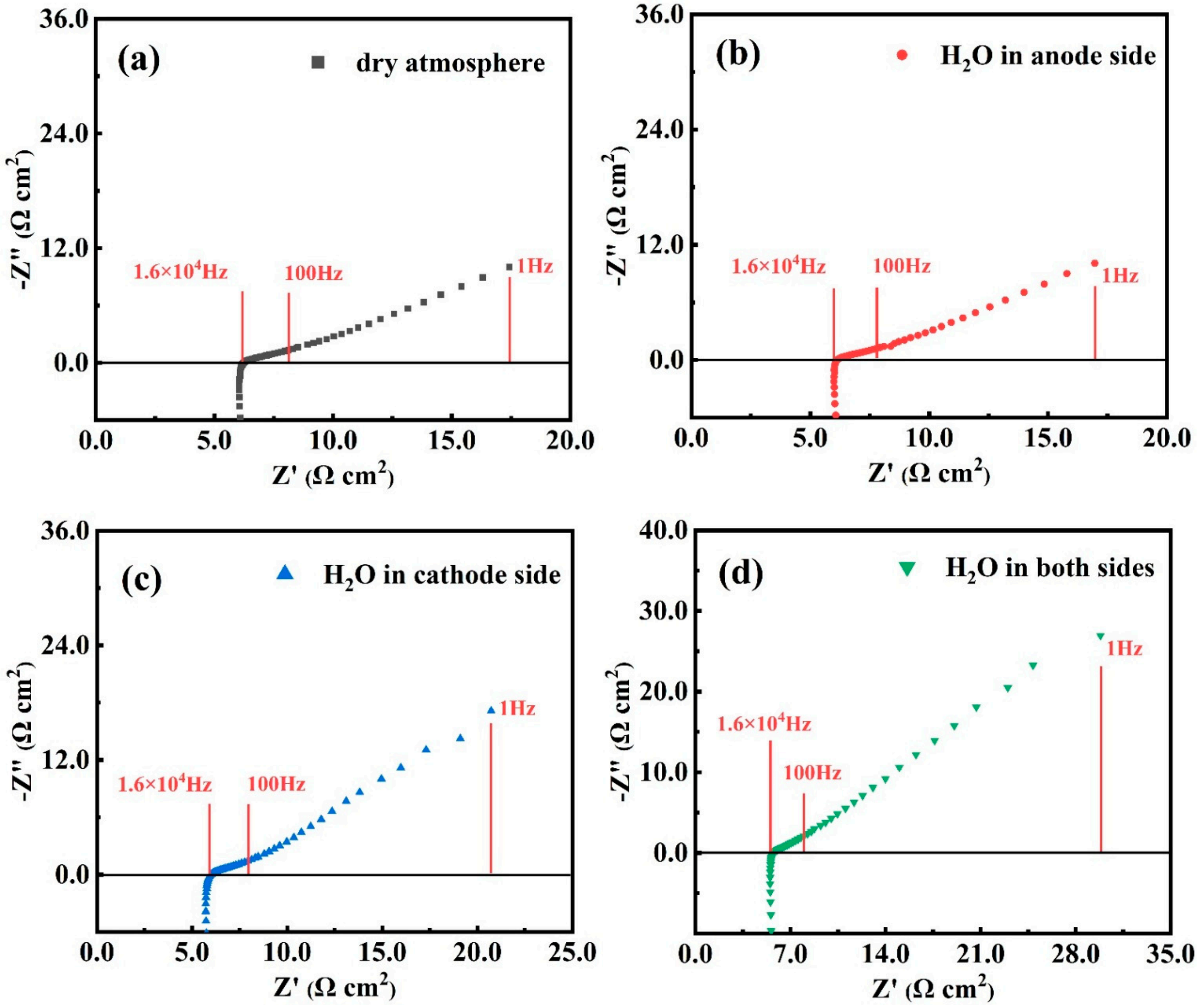

Figure S1. Impedance spectra measured under 0.5 V at 600 °C in a H_2 atmosphere with water vapor supplied at different positions, recorded from high to low frequency. (**a**) dry atmosphere; (**b**) water vapor is applied to the anode side; (**c**) water vapor is applied to the cathode side; (**d**) water vapor is applied to both sides.

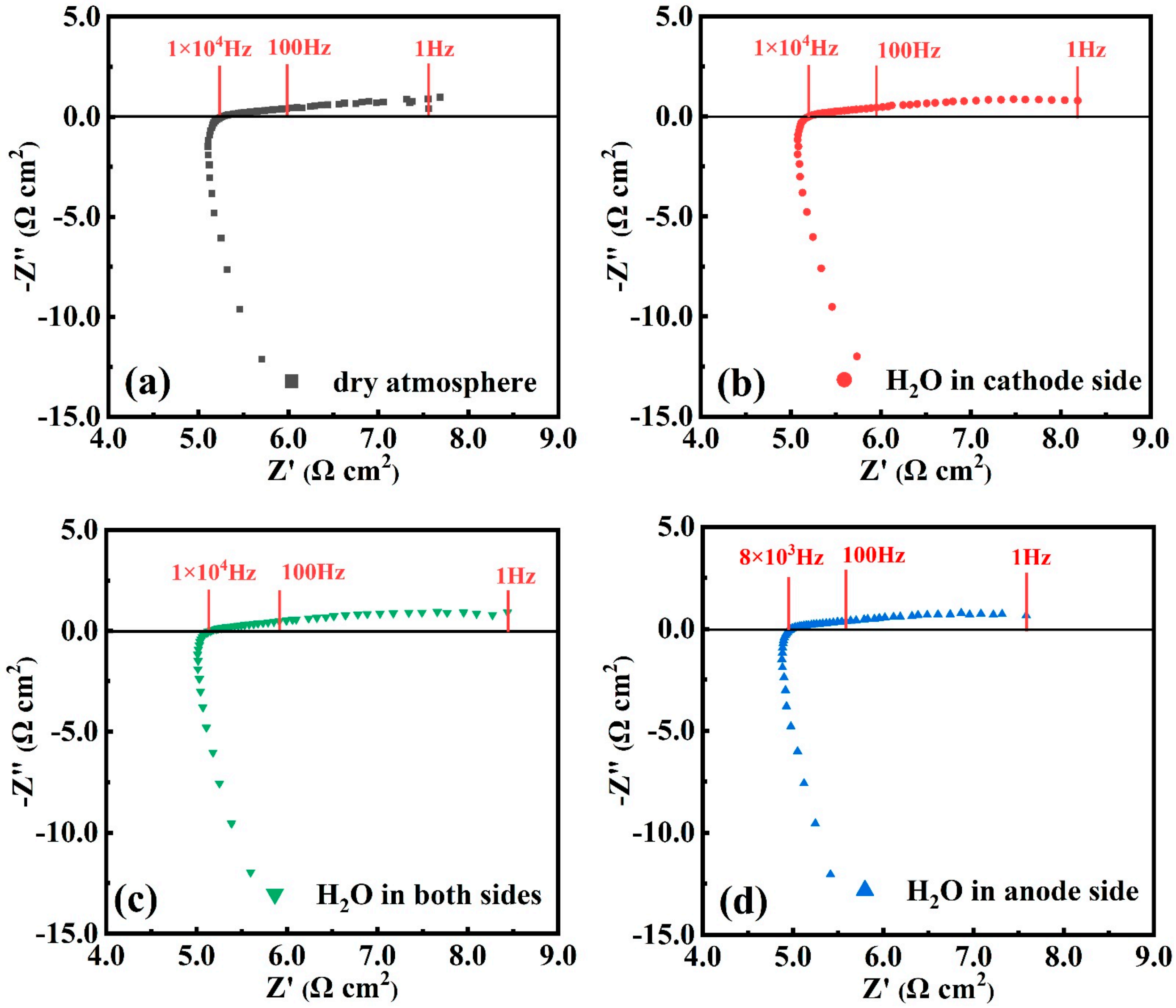

Figure S2. Impedance spectra measured under 0.5 V at 600 °C in a O_2 atmosphere with water vapor supplied at different positions, recorded from high to low frequency. (**a**) Dry atmosphere; (**b**) water vapor is applied to the cathode side; (**c**) water vapor is applied to both sides; (**d**) water vapor is applied to the anode side.
